# Palpitation was associated with clinical outcomes in patients with hypertrophic cardiomyopathy

**DOI:** 10.1038/s41598-020-71797-y

**Published:** 2020-09-10

**Authors:** Tingting Hu, Tao Wang, Xiwen Zhang

**Affiliations:** 1 Department of Cardiology, The Affiliated Huaian No.1 People’s Hospital of Nanjing Medical University, Beijing West Road 6, Huai’an, 223001 China; 2 Jiangsu College of Nursing, Huai’an, 223001 China

**Keywords:** Cardiology, Diseases

## Abstract

Hypertrophic cardiomyopathy (HCM) is a common genetic heart disease with diversified clinical presentation and it is important to identify new predictors of clinical outcomes and survival in HCM patients. In our study, 206 HCM patients were compared with respect to major adverse cardiovascular and cerebrovascular events. By multivariable logistic analysis, we determined that palpitation, together with chronic heart failure (CHF) > 1 year, was an independent predictor of major adverse cardiovascular and cerebral events (MACCE) in HCM patients (OR 3.24, 95% CI 1.60–6.57, P = 0.001). Specially, palpitation was related to higher prevalence of rehospitalization (OR 3.86, 95% CI 2.08–7.08, P < 0.001), cardiac death (OR 2.96, 95% CI 1.05–8.32, P = 0.04) and heart failure exacerbation (OR 4.07, 95% CI 2.04–8.13, P < 0.001). However, patients presented with palpitation did not show a significantly different cardiac phenotype and function. Finally, palpitation predicted a poor prognosis in HCM patients without atrial fibrillation by utilizing Kaplan–Meier analysis (P = 0.041). In conclusion, palpitation could be a new predictor of clinical outcomes and overall survival in HCM patients.

## Introduction

Hypertrophic cardiomyopathy is a common genetic heart disease, with a incidence of more than 1/500^1^. The reported annual mortality risk is variable, more than 1.5% in population-based cohorts^2^. The genetic studies have demonstrated that HCM is caused by mutation of at least 11 genes encoding thick and thin contractile myofilament of the sarcomere or adjacent Z discs^3^. For 2 decades, more than 1,400 mutations have been identified, among which MYH7, MYBPC3 and TNNT2 accounted for 70% of HCM patients^4^. The clinical consequences of HCM patients varied from asymptomatic to diastolic dysfunction, progressive heart failure, atrial fibrillation, stroke and cardiac death^5^.

Due to genetic and phenotypic heterogeneity of HCM patients, the correlation between sarcomere mutation and clinical outcomes is unstable^6^, resulting in the inability of specific gene mutations to determine the prognosis^7^. Interplay of modifier genes and environmental factors is considered to be a potential explanation for phenotypic diversity^8^. Long-term efforts have focused on the survival and prognostic factors in HCM, such as age, family history, non-sustained ventricular tachycardia, obstruction and so on^9,10^. Therefore, it is very important to search for the better prognostic factors of HCM patients from their clinical characteristics.

Our study is to describe the presence and prognostic effect of palpitation on MACCE of HCM patients. Besides, we also investigated the association between palpitation and overall survival of HCM without or with atrial fibrillation (AF).

## Results

### Clinical characteristics of HCM patients

The study population consisted with 206 HCM patients, and was divided into 2 groups based on the presence of MACCE. Demographic and clinical characteristics of the two groups were shown in Table 1, including 57 cases (27.7%) with MACCE and 149 cases (72.3%) without MACCE.

**Table 1:** Clinical, electrocardiographic and echocardiographic features of the 206 HCM patients with or without MACCE.

	All patients (206)	MACCE (57)	non-MACCE (149)	P
**Demographics**				
Age (year)	61.1 ± 12.3	64.3 ± 14.1	59.9 ± 12.4	0.633
Men	131 (63.6)	28 (49.1)	103 (69.1)	0.008
BMI(g/m^2^)	24.9 ± 3.3	25.0 ± 3.7	24.5 ± 3.2	0.125
Family history	8 (3.9)	3 (5.3)	5 (3.4)	0.526
CAD	33 (16.0)	8 (14.0)	25 (16.8)	0.631
Hypertension	117 (56.8)	36 (63.2)	81 (54.4)	0.254
CHF > 1 year	16 (7.8)	12 (21.1)	4 (2.7)	< 0.001
NYHA class III or IV	21 (10.2)	14 (24.6)	7 (4.7)	< 0.001
sBP(mmHg)	133.5 ± 22.8	130.6 ± 24.6	131.1 ± 21.1	0.239
dBP(mmHg)	75.5 ± 13.7	75.4 ± 14.4	75.7 ± 13.2	0.145
**Clinical presentations**				
Palpitation	66 (32.4)	31 (54.4)	35 (23.8)	< 0.001
Syncope	17 (8.3)	7 (12.3)	10 (6.8)	0.209
Chest pain	51 (25.1)	11 (19.3)	40 (27.4)	0.232
**Medical treatment**				
Beta-blockers	169 (82.0)	50 (87.7)	119 (79.9)	0.189
ACEI/ARB	58 (28.2)	17 (29.8)	41 (27.5)	0.742
Diuretics	19 (9.2)	9 (15.8)	10 (6.7)	0.044
Aspirin	97 (47.1)	27 (47.4)	70 (47.0)	0.960
Wafarin	28 (13.6)	10 (17.5)	18 (12.1)	0.306
Amiodarone	11 (5.3)	3 (5.3)	8 (5.4)	0.976
ICD	20 (9.7)	9 (15.8)	11 (7.4)	0.068
**Electrocardiography**				
HR (bpm)	68.4 ± 11.6	72.3 ± 17.0	72.4 ± 18.8	0.764
PR duration (ms)	171.3 ± 27.9	170.9 ± 31.9	172.4 ± 30.2	0.682
QRS duration (ms)	101.6 ± 16.5	106.6 ± 28.0	101.2 ± 18.1	0.025
QTc (ms)	437.4 ± 22.2	439.5 ± 28.5	434.1 ± 24.8	0.843
LV hypertrophy	119 (57.8)	33 (57.9)	86 (57.7)	0.982
T inversion	19 (9.2)	7 (12.3)	12 (8.1)	0.348
QRST angle > 90°	141 (68.4)	38 (66.7)	103 (69.1)	0.734
BBB	15 (7.3)	3 (5.4)	12 (8.1)	0.509
Atrial fibrillation	55 (26.7)	18 (31.6)	37 (24.8)	0.327
**Echocardiography**				
LVDd (cm)	4.90 ± 0.42	4.97 ± 0.64	4.85 ± 0.49	0.020
LVDs (cm)	3.34 ± 0.35	3.42 ± 0.50	3.33 ± 0.41	0.083
IVSTd (cm)	1.53 ± 0.46	1.53 ± 0.39	1.52 ± 0.47	0.059
LVPWTD (cm)	1.15 ± 0.23	1.19 ± 0.23	1.16 ± 0.32	0.627
LAD (cm)	4.28 ± 0.54	4.52 ± 0.54	4.31 ± 0.58	0.580
**Thickening site**				0.586
Septum	37 (18.0)	9 (17.0)	28 (19.4)	
Left ventricle	111 (53.9)	33 (62.3)	78 (54.2)	
Apex	49 (23.8)	11 (20.8)	38 (26.4)	
Max thickening (cm)	1.70 ± 0.35	1.84 ± 0.43	1.77 ± 0.35	0.499
LVM (g)	323.2 ± 101.9	330.6 ± 99.0	315.6 ± 108.3	0.314
LVMI (g/m^2^)	184.2 ± 56.6	192.0 ± 56.6	179.8 ± 57.9	0.799
LVEF (%)	60.8 ± 17.2	56.6 ± 7.3	59.9 ± 17.0	0.834
LV systolic dysfunction	13 (6.3)	6 (10.9)	7 (4.8)	0.113
E/A	0.99 ± 0.44	1.05 ± 0.48	0.95 ± 0.42	0.085
Obstruction	49 (23.8)	14 (24.6)	35 (23.5)	0.872

*BMI* body mass index, *CAD* coronary artery disease, *CHF* chronic heart failure, *sBP* systolic blood pressure, *dBP* diastolic blood pressure, *ACEI/ARB* angiotensin converting enzyme inhibitors and angiotensin II receptor blockers, *ICD* implantable cardioverter defibrillator, *LVEDd* left ventricular end diastolic diameter, *LVEDs* left ventricular end systolic diameter, *IVSTD* end diastolic ventricular septal thickness, *LVPWTD* left ventricular posterior wall end diastolic thickness, *AoD* aortic dimension, *LAD* left atrium diameter, *LVEF* left ventricular ejection fraction, *LVM* left ventricular mass, *LVMI* left ventricular index, *MACCE* major adverse cardiovascular and cerebral events.

### Comparisons between the MACCE and non-MACCE group in HCM patients

As shown in Table 1, there were no significant differences in age, BMI, family history, blood pressure, as well as presence of CAD and hypertension. Compared with non-MACCE group, MACCE group did not significantly differ with respect to clinical presentations other than palpitation (P < 0.001), as well as medical treatments but diuretics (P = 0.044). Accordingly, electrocardiographic and echocardiographic index were highly in the MACCE group, but there was no statistical difference except for QRS duration (P = 0.025) and LVDd (P = 0.020). MACCE patients showed higher prevalence of CHF > 1 year (P < 0.001) and NYHA class III or IV (P < 0.001).

### Multivariable logistic regression analyses to identify independent determinants of MACCE in HCM patients

Multivariable analyses indicated that only palpitation and CHF > 1 year were independent determinants of MACCE in HCM patients (Table 2), whereas gender, NYHA class III or IV, diuretics, QRS duration and LVDd were not. The model indicated that HCM patients presented with palpitation were 3.24 times more likely to develop MACCE than those without palpitation (OR 3.24, 95% CI 1.60–6.57, P = 0.001).

**Table 2:** Multivariable logistic regression analysis of HCM patients with MACCE.

	OR	95% CI	P
CHF > 1 year	10.65	3.09–16.79	< 0.001
Palpitation	3.24	1.60–6.57	0.001

*MACCE* major adverse cardiovascular and cerebral events, *CHF* chronic heart failure.

### Relation of palpitation to LV mass and function or other clinical variables

To exclude the impact of palpitation on the LV mass and function, which were closely related to clinical outcomes of HCM patients, we explored the difference in LVM and function between two groups. Average LVM and LVMI in the HCM patient cohort were 323.2 ± 101.9 g/m^2^ and 184.2 ± 56.6 g/m^2^ respectively. Compared with non-MACCE group, LVM, LVMI and LVEF were decreased in MACCE cohorts, but there was no statistic difference (Supplementary Fig. 1). Besides, MACCE patients has a significantly higher E/A ratio that non-MACCE patients (P = 0.002). Besides, we further described the characteristics of patients according to the presence of palpitation. We found that clinical, electrocardiographic and echocardiographic features were comparable in patients with or without palpitation (Supplementary Table 1). These results suggested that palpitation was not associated with LV mass, function or other clinical variables.

### Palpitation and clinical outcome in HCM patients

To specifically assess the influence of palpitation on clinical outcomes in HCM cohorts, we compared the relative risk of rehospitalization, cardiac death, heart failure exacerbation and stroke between palpitation group and non-palpitation group. From Table 3, the HCM patients with palpitation had highly presence of rehospitalization (OR 3.86, 95% CI 2.08–7.08, P < 0.001), cardiac death (OR 2.96, 95% CI 1.05–8.32, P = 0.04) and heart failure exacerbation (OR 4.07, 95% CI 2.04–8.13, P < 0.001). However, no difference was observed for stroke between two groups.

**Table 3:** The association between palpitation and clinical outcomes of HCM patients.

	Non palpitation	Palpitation	OR	95% CI	P
Rehospitalization	45 (32.6)	43 (65.2)	3.86	2.08–7.08	< 0.001
Cardiac death	7 (5.1)	9 (13.6)	2.96	1.05–8.32	0.04
Heart failure exacerbation	19 (13.8)	26 (39.4)	4.07	2.04–8.13	< 0.001
Stroke	4 (2.9)	2 (3.0)	1.05	0.19–5.87	0.958

### The impact of palpitation on overall survival of HCM patients with or without atrial fibrillation (AF)

**Figure 1:**
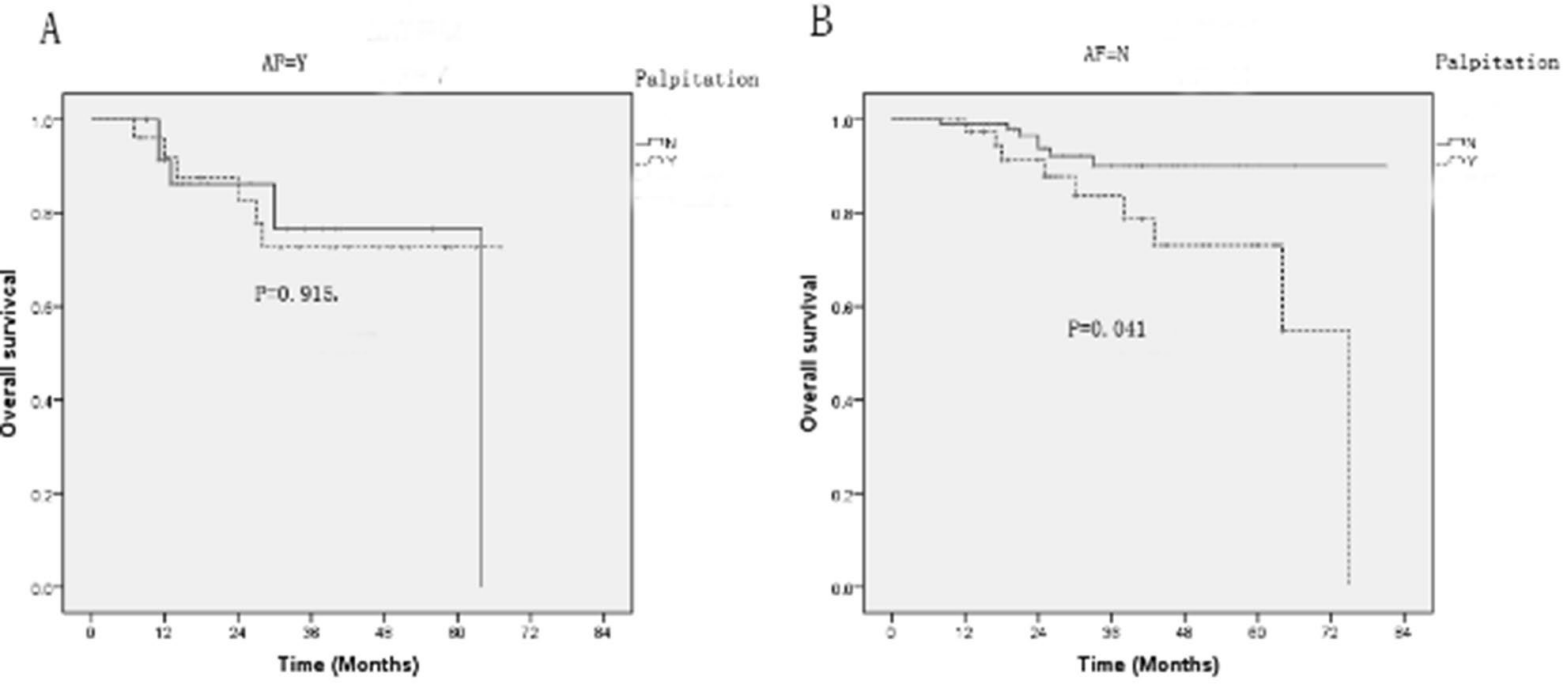
The survival analysis of palpitation in HCM patients with or without AF. (**A**) The HCM cohorts with AF showed no significant difference in overall survival. (**B**) For HCM patients without AF, patients with palpitation had a poor survival that without palpitation. *AF* atrial fibrillation.

For all HCM patients, there was no significant difference in overall survival between two groups (Fig. 1). Palpitation is an important clinical manifestation of AF, which affects the quality of life of HCM patients and predicts poor prognosis^11^. Therefore, we investigated the relationship between palpitation and overall survival rate of HCM patients stratified by AF. Compare with HCM patients without palpitation, palpitation patients had a poor survival among HCM patients without atrial fibrillation (P = 0.041). However, the poor prognosis of palpitation was not statistically significant in HCM patients with atrial fibrillation.

## Discussion

We reported that palpitation contributed to MACCE in HCM patients, independent on LV mass and LV function. Palpitation was also associated with increased risk of rehospitalization, cardiac death and heart failure exacerbation. Our novel findings were that HCM patients with palpitation have a more poor survival compared with patients without palpitation, but the prognosis of palpitation is only observed in non-AF populations, with no statistical difference in AF populations.

From Table 1, we found that MACCE patients differed in CHF > 1 year (P < 0.001) and NYHA class III or IV (P < 0.001) from non-MACCE cohorts. For a series of common cardiovascular risk, we did not observe significant difference in age, BMI, family history, blood pressure, as well as presence of CAD and hypertension between two groups. Many electrocardiographic and echocardiographic index were not significantly different other than QRS duration (P = 0.025) and LVDd (P = 0.020). Because CHF > 1 year and NYHA class III or IV reflected cardiac function and were closely related to adverse cardiovascular events^12,13^. So, a multivariable logistic regression model was constructed only with variables that proved significant at univariate analysis. From Table 2, we concluded that only CHF > 1 year, NYHA class III or IV and palpitation were independently associated with MACCE of HCM patients. Strikingly, patients presented with palpitation had a 3.24-time risk to develop MACCE than those without palpitation. To confirm whether worse cardiovascular outcomes caused by palpitation were attributed to a poor cardiac phenotype and function^14,15^, we compared the LV mass, LV mass index, LV systolic and LV diastolic function between palpitation groups and non-palpitation groups, which showed no statistical difference between two groups. These results suggested that palpitation on HCM patients was not related to the increased LV mass and decreased LV function. As shown in Table 3, the HCM patients with palpitation had higher risk of rehospitalization, cardiac death and heart failure exacerbation. Although the occurrence of stroke was relatively higher in patients with palpitation than that without palpitation, there was no significant difference. Kaplan–Meier survival analysis revealed that palpitation did not have an obvious effect on overall survival in HCM patients. Considering that AF is common and bears a major clinical impact in HCM patients^16^, we explored the association of palpitation in patients in the absence of AF. Interestingly, palpitation predicted a poor prognosis among HCM patients without AF.

Our study showed that palpitation was related to more risk of MACCE in HCM patients, including rehospitalization, cardiac death and heart failure exacerbation, which was independent on LV mass and LV function, and had a poor prognosis on overall survival in HCM patients without AF. These results indicated that palpitation could become a new predictor of MACCE in HCM patients and overall survival of HCM cohorts without AF. Some palpitations may be an indication of cardiac arrhythmias^17^, which influence the prognosis of HCM patients. Besides, some benign palpitation could be psychiatric, causing considerable distress and disability of HCM patients^18^. However, the underlying mechanism between palpitation and MACCE of HCM patients needs to be further investigated.

This study had several limitations. This was a cross-sectional study, which includes a long-term follow-up about all-cause death. Although there was obvious difference in clinical outcomes of HCM patients between two groups, the exact causes of adverse cardiac events could not be analyzed. Besides, palpitation was a self-reported clinical presentation, which may not be definitively objective. In addition, we did not have cardiac magnetic resonance data, which may facilitate accurate diagnosis of HCM compared to echocardiography.

In conclusion, our study demonstrated that palpitation increased the risk of MACCE in HCM patients, which was independent of LV mass and LV function, and had a poor prognosis on overall survival in HCM patients without AF. These results indicated that palpitation could become a new predictor of MACCE in HCM patients and overall survival of HCM cohorts.

## Methods

### Study population

Patients who were admitted for HCM in the department of cardiology at Huaian No.1 People’s Hospital from January 2010 to December 2015 were retrospectively recruited in this study. 206 patients with complete follow-up data by June 2018 were enrolled in this study. The criteria of a diagnosis of HCM was in accordance with 2014 European Society of Cardiology guidelines on diagnosis and management of hypertrophic cardiomyopathy^19^. It was defined as unexplained LV hypertrophy with a maximal wall thickness above 15 mm showed by any cardiac imaging, such as echocardiography, cardiac magnetic resonance or computed tomography. 206 patients which were admitted to cardiology department from outpatient were all subject to echocardiography, with some subjected to additional echocardiography. All patients with cardiac hypertrophy caused by physiologic cardiac hypertrophy, rheumatic heart disease, mitral valve disease, amyloidosis and Fabry disease were excluded, and some HCM patients with history of renal disease, cancer disease or acute coronary syndrome with cardiogenic shock were also excluded. Palpitations were described as flip-flopping in the chest, rapid fluttering in the chest, and pounding in the neck, which occurred more than once a month and lasted for at least 10 min^20,21^. The patients were excluded for those with cardiac neurosis, supraventricular arrhythmias, ventricular tachycardia and other arrhythmias except for atrial fibrillation, and they do not have hemodynamic compromises. The family history referred to the first-degree relatives suffering HCM or sudden cardiac death. Patients with the symptom of dyspnea, wheezing, or edema (New York Heart Association [NYHA] functional class II, III, or IV) for more than 1 year were defined as CHF > 1 year. Notably, the age was referred to the time of the HCM patients on admission, not at the first diagnosis. The primary endpoint was MACCE, including cardiac death, heart failure rehospitalization and stroke. This study was approved by Huaian No.1 People’s Hospital Review Board.

### Cardiac evaluation

Electrocardiographic and echocardiography studies were performed with commercially available instruments. Electrocardiographic data included heart rate, PR duration, QRS duration, QTc and QRST angle. And echocardiographic data included left ventricular end diastolic diameter (LVEDd), left ventricular end systolic diameter (LVEDs), end diastolic ventricular septal thickness (IVSDD), left ventricular posterior wall end diastolic thickness (LVPWTD), aortic dimension (AoD), left ventricular ejection fraction (LVEF) and E/A ratio. Left ventricular outflow obstruction was identified by a peak instantaneous outflow gradient ≥ 30 mmHg occurring under basal conditions. LV systolic dysfunction was defined as LVEF < 50%. Left ventricular mass (LVM) and left ventricular mass index (LVMI) were calculated based on the Devereux method.

### Statistical analysis

Continuous variables were expressed as mean ± SD, and categorical variables were expressed as a proportion. For the comparison of normally distributed variables, we employed Student t test. Chi-square test was utilized to compare categorical variables; Fisher exact test was employed when 1 or more cells in the comparison table had an expected frequency of < 5. The dependence of variables associated with increased MACCE were assessed by multivariable logistic regression analysis, which was constructed only with clinical features that proved significant at univariate analysis. Survival analysis was described by using the Kaplan–Meier survival analysis method with a log-rank test. P values are 2-sided and considered significant when < 0.05. The statistical analysis was carried out with the use of SPSS 12.0 software (Chicago, Illinois).

### Ethics approval

This study was approved by Huaian No.1 People’s Hospital Review Board.

### Consent to participate

The patients provided their written informed consent to participate in this study.

## Supplementary information

Supplementary Legend.

Supplementary Figure 1.

Supplementary Table 1.

## Abbreviations

AF: Arterial fibrillation
CAD: Coronary arterial disease
CHF: Chronic heart failure
HCM: Hypertrophic cardiomyopathy
LVM: Left ventricular mass
LVMI: Left ventricular mass index
LVEF: Left ventricular ejection fraction
MACCE: Major adverse cardiovascular and cerebrovascular events
NYHA: New York Heart Association
