# Supplementary material for: Palpitation was associated with clinical outcomes in patients with hypertrophic cardiomyopathy
Source: Sci Rep. 2020 Sep 10;10:14935. doi: 10.1038/s41598-020-71797-y (PMC7483715; doi:10.1038/s41598-020-71797-y)
Supplement: Supplementary file 1 — Supplementary Legend. [file 41598_2020_71797_MOESM1_ESM.docx]

**Supplementary Figure 1. The relation of palpitation with LV phenotype and function. (A) LV mass and LV mass index showed no significant difference between palpitation and non-palpitation groups. (B) The LV function of palpitation cohorts was not better than that of non-palpitation cohorts.**
