# Supplementary figures and images for: Palpitation was associated with clinical outcomes in patients with hypertrophic cardiomyopathy
Source: Sci Rep. 2020 Sep 10;10:14935. doi: 10.1038/s41598-020-71797-y (PMC7483715; doi:10.1038/s41598-020-71797-y)

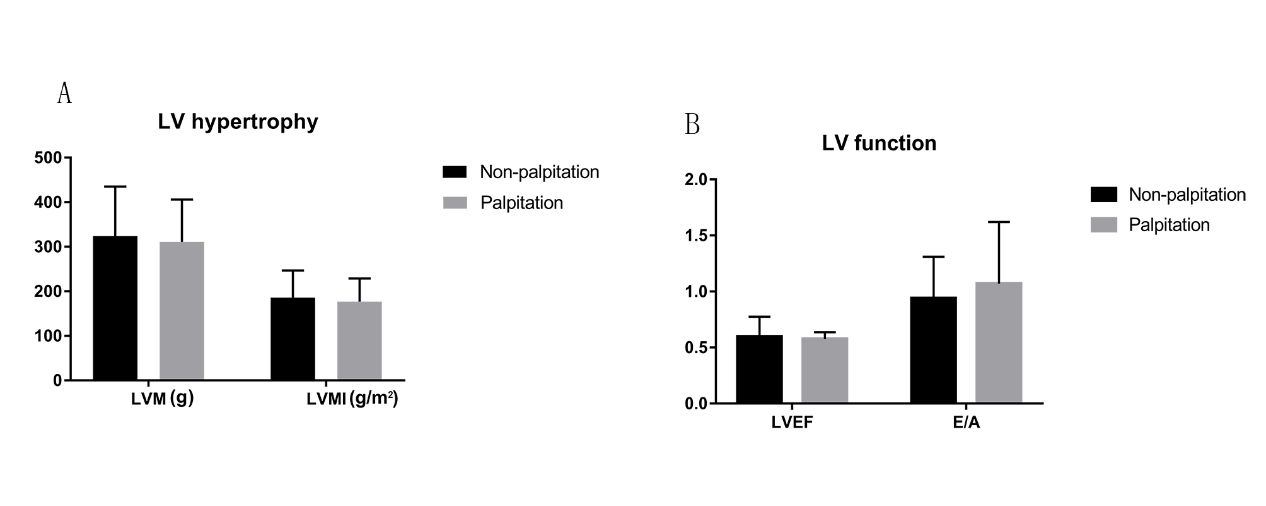

Supplement: Supplementary file 2 — Supplementary Figure 1. [file 41598_2020_71797_MOESM2_ESM.tif]
