# Supplementary material for: Palpitation was associated with clinical outcomes in patients with hypertrophic cardiomyopathy
Source: Sci Rep. 2020 Sep 10;10:14935. doi: 10.1038/s41598-020-71797-y (PMC7483715; doi:10.1038/s41598-020-71797-y)
Supplement: Supplementary file 3 — Supplementary Table 1. [file 41598_2020_71797_MOESM3_ESM.docx]

**Supplementary Table 1. The clinical, electrocardiographic and echocardiographic features of the 206 HCM patients with or without palpitation.**

|  | All patients(206) | | Palpitation (66) | non-palpitation (140) | | | P value |  |
| --- | --- | --- | --- | --- | --- | --- | --- | --- |
| Demographics |  |  | | |  |  | | |
| Age(yr) | 61.1±12.3 | 62.3±12.1 | | | 60.5.9±12.4 | 0.831 | | |
| Men | 131(63.6) | 35(53.0) | | | 96 (68.6) | 0.044 | | |
| BMI(g/m2) | 24.9±3.3 | 25.8±3.2 | | | 24.5±3.3 | 0.885 | | |
| Family history | 8(3.9) | 2(3.0) | | | 6(4.3) | 0.961 | | |
| CAD | 33(16.0) | 10(15.1) | | | 23(16.4) | 0.976 | | |
| Hypertension | 117(56.8) | 40(60.6) | | | 77(55.0) | 0.544 | | |
| CHF>1yr | 16(7.8) | 11(16.7) | | | 5(3.6) | 0.003 | | |
| NYHA class III or IV | 21(10.2) | 15(22.7) | | | 6(4.3) | <0.001 | | |
| sBP(mmHg) | 133.5±22.8 | 131.5±25.1 | | | 134.4±21.7 | 0.153 | | |
| dBP(mmHg) | 75.5±13.7 | 76.3±14.9 | | | 75.1±13.1 | 0.730 | | |
|  |  |  | | |  |  | | |
| Clinical presentations |  |  | | |  |  | | |
| Syncope | 17(8.3) | 9(13.6) | | | 8(5.7) | 0.098 | | |
| Chest pain | 51(25.1) | 13(19.7) | | | 38(27.1) | 0.326 | | |
|  |  |  | | |  |  | | |
| Medical treatment |  |  | | |  |  | | |
| beta-blockers | 169(82.0) | 55(83.3) | | | 114(81.4) | 0.326 | | |
| ACEI/ARB | 58(28.2) | 30(30.3) | | | 38(27.1) | 0.761 | | |
| Diuretics | 19(9.2) | 10(15.2) | | | 0(6.4) | 0.078 | | |
| Aspirin | 97(47.1) | 30(45.5) | | | 67(47.9) | 0.863 | | |
| Wafarin | 28(13.6) | 8(12.1) | | | 20(14.3) | 0.838 | | |
| Amiodarone | 11(5.3) | 4(6.1) | | | 7(5.0) | 0.999 | | |
| ICD | 20(9.7) | 8(12.1) | | | 12(8.6) | 0.582 | | |
|  |  |  | | |  |  | | |
| Electrocardiography |  |  | | |  |  | | |
| HR (bpm) | 68.4±11.6 | 74.1±15.2 | | | 65.7±9.9 | 0.999 | | |
| PR duration(ms) | 171.3±27.9 | 169.8±25.4 | | | 172.0±29.1 | 0.277 | | |
| QRS duration(ms) | 101.6±16.5 | 104.8±27.4 | | | 100.1±11.4 | 0.996 | | |
| QTc (ms) | 437.4±22.2 | 440.5±29.1 | | | 435.9±18.9 | 0.963 | | |
| LV hypertrophy | 119(57.8) | 33(53.9) | | | 84(60.7) | 0.427 | | |
| T inversion | 19(9.2) | 8(12.1) | | | 11(7.9) | 0.466 | | |
| QRST angle>90° | 141(68.4) | 40(60.6) | | | 101(72.1) | 0.133 | | |
| BBB | 15(7.3) | 4(6.1) | | | 11(7.9) | 0.861 | | |
| Atrial fibrillation | 55(26.7) | 20(30.3) | | | 35(25.0) | 0.526 | | |
|  |  |  | | |  |  | | |
| Echocardiography |  |  | | |  |  | | |
| LVDd(cm) | 4.90±0.42 | 4.91±0.56 | | | 4.90±0.35 | 0.506 | | |
| LVDs(cm) | 3.34±0.35 | 3.40±0.56 | | | 3.31±0.25 | 0.533 | | |
| IVSTd(cm) | 1.53±0.46 | 1.51±0.38 | | | 1.54±0.50 | 0.489 | | |
| LVPWTD(cm) | 1.15±0.23 | 1.21±0.31 | | | 1.12±0.19 | 0.534 | | |
| LAD(cm) | 4.28±0.54 | 4.40±0.31 | | | 4.22±0.65 | 0.568 | | |
| Thickening site |  |  | | |  | 0.849 | | |
| septum | 37(18.0) | 10(15.2) | | | 27(19.3) |  | | |
| left ventricle | 111(53.9) | 35(53.0) | | | 76(54.3) |  | | |
| apex | 49(23.8) | 14(21.2) | | | 35(25.0) |  | | |
| Max thickening(cm) | 1.70±0.35 | 1.81±0.50 | | | 1.65±0.28 | 0.563 | | |
| LVM(g) | 323.2±101.9 | 315.3±98.3 | | | 326.9±103.6 | 0.183 | | |
| LVMI(g/m2) | 184.2±56.6 | 181.4±57.0 | | | 190.1±55.8 | 0.888 | | |
| LVEF (%) | 60.8±17.2 | 58.8±10.1 | | | 61.7±20.5 | 0.141 | | |
| LV systolic dysfunction | 13(6.3) | 5(7.6) | | | 8(5.7) | 0.837 | | |
| E/A | 0.99±0.44 | 1.04±0.48 | | | 0.97±0.42 | 0.529 | | |
| Obstruction | 49(23.8) | 15(22.7) | | | 34(24.3) | 0.944 | | |
